# Exploring the relationship between postnatal depressive symptoms and parental burnout from the perspective of the population and individual level

**DOI:** 10.1186/s12888-023-04853-2

**Published:** 2023-06-07

**Authors:** Yongqi Huang, Fangxiang Mao, Xuan Zhang, Juan wang, Zhaojuan Xu, Fenglin Cao

**Affiliations:** 1 School of Nursing and Rehabilitation, Shandong University, No.44 Wenhua West Road, 250012 Jinan, Shandong China; 2 The Second Affiliated Hospital of Shandong University, Jinan, Shandong Province China

**Keywords:** Postnatal depressive symptoms, Parental burnout, Latent class analysis, Postnatal mothers

## Abstract

**Background:**

Parental burnout has become increasingly common, which can lead to a range of adverse outcomes. Postnatal mothers are vulnerable and mothers with high postpartum depression scores may be more prone to parental burnout. This study aims to investigate the association between postnatal depressive symptoms and parental burnout at both the population and individual levels.

**Methods:**

This study comprised a cross-sectional study design and participants were recruited using convenience sampling. A total of 560 postnatal mothers answered a questionnaire on their general information, postnatal depressive symptoms and parental burnout. Multiple linear regression and binary logistic regression analysis were used to examine the association between postnatal depressive symptoms and parental burnout. Furthermore, latent class analysis was used to identify subtypes of parental burnout. Finally, binary logistic regression was used to examine the differences in postnatal depressive symptoms between latent classes comprising parental burnout.

**Results:**

The prevalence of burnout was approximately 10%. At the population level, postnatal depressive symptoms were positively associated with parental burnout (all *P* < 0.05). At the individual level, two latent classes were identified (i.e., “low parental burnout class” and “high parental burnout class”). Moreover, mothers with postnatal depressive symptoms were more likely to be associated with high parental burnout (PB) class than the low parental burnout class (*OR* = 1.12, 95% CI:1.03 to 1.23).

**Conclusion:**

This study found a positive relationship between postnatal depressive symptoms and parental burnout. It provided evidence for developing depression-targeted programs for parental burnout, which could bring great benefits for both mothers and infants.

**Supplementary Information:**

The online version contains supplementary material available at 10.1186/s12888-023-04853-2.

## Introduction

While parenthood is a joyful event, it places pressure on mothers and may even lead to parental burnout. Parental burnout is common. It is estimated that approximately 20% of mothers experiences parental burnout [1]. The adverse effects of parental burnout are considerable. It may not only bring about addictive behaviours, sleep problems, and suicidal ideation in mothers themselves, but also may damage couple relationships and increase the risk of maladaptive parenting behaviour (e.g., neglect, violence) [2].

Postnatal mothers are vulnerable for parental burnout. This may result from special challenges during this period [3]. For example, the increasing psychosomatic discomforts during postnatal period, substantial acute (e.g., infant’s illness, falls) and chronic parenting stressors (e.g., difficult infant temperament) from infants [4]. Therefore, it is necessary to consider parental burnout among postnatal mothers.

Among the influencing factors mentioned above, maternal postnatal depressive symptoms could be an important factor. The severity and specialty of postnatal depression have been recognized worldwide. In China, 14.8% of mothers suffer from postnatal depression [5]. Postnatal depression not only affects the mother’s social and occupational functioning [6], but also interferes with the mother-infant relationship and impairs the cognitive, behavioral, and social-emotional development of the offspring[7]. Considering the adverse effects of postnatal depression [8], timely and early treatment of maternal depression is important to protect the health of the mother and offspring(e.g. electroconvulsive therapy) [9], and studies have found that if left untreated it can lead to poorer parenting behaviors and thereby increased parental burnout [10]. Limited studies have underscored the relationship between maternal depressive symptoms and parental burnout [1, 11–13]. However, previous studies mostly focused on mothers with older children. Exploring the association between postnatal depressive symptoms and parental burnout during one year postpartum could provide evidence for developing depression-targeted programs for parental burnout, which could bring great benefits for both mothers and infants. In addition, previous studies used non-specialized parental burnout measures, like BMI-10 [14], to measure parental burnout, which may lead to inexact estimation about the severity of parental burnout and further bias the association between depression symptoms and parental burnout [1, 12, 13]. Furthermore, a few recent studies have indicated the heterogeneity of parental burnout [15, 16]. For example, Lebert-Charron et al. identified five clusters based on parental burnout symptoms in French mothers [15]. The results further showed that affective variables (e.g., anxiety, depressive symptoms, and burden) differed between the clusters. However, this study only used ANOVA for comparisons between clusters, which ignored the effect of confounding factors.

Ignoring individual heterogeneity may hinder the ability to distinguish between groups with different characteristics in a population. Latent Class Analysis (LCA) is a person-centered analysis method that determines the potential characteristics of individuals for classification based on their classification on dimensions of scale, identifies groups with different characteristics in the population, maximizes the differences between groups, minimizes the differences within groups, and allows the accuracy and validity of the classification to be assessed using objective fit indicators [17]. The categories recognized by mothers are based on the level of manifestations of parental burnout at the individual level. Thus, clinicians are able to identify mothers experiencing significant challenges, promote specific primary prevention, and advise on targeted therapeutic interventions for parental burnout [15]. This study aimed to investigate the association between postnatal depressive symptoms and parental burnout at both the population and individual levels.

## Methods

This study comprised a cross-sectional study design. Using a convenience sampling, participants were recruited from a pediatric health clinic at a tertiary care hospital in Jinan, China, between October 2020 and October 2021.

### Participants

The inclusion criteria were as follows: (1) within a year postpartum, (2) age ≥ 18 years at the time of conception, (3) natural pregnancy, (4) singleton pregnancy, (5) full-term delivery, (6) normal verbal communication and comprehension skills, as well as ability to read and complete the questionnaire correctly. Patients with (1) severe psychiatric disorders, (2) severe physical diseases, or (3) a baby diagnosed with a congenital or severe disease were excluded from the study.

### Sample size caculation

In the multiple linear regression, the expected effect size (f^2^) was taken as a medium effect size of 0.15, the statistical efficacy was taken as 90%, the significance level was set at 0.05, and the estimated number of predictor variables was 13. In G*power, the minimum sample size required for the calculation was 164. Considering a null response rate of approximately 20%, the minimum sample size required was 208. Empirically, a minimum of 300 cases was required to identify the optimal number of potential class models [18], and considering a null response rate of approximately 20%, the minimum sample size required was 375. In summary, the minimum required sample size was 375.

### Measures

#### Parental burnout assessment (PBA)

The PBA was used to measure the level of parental burnout [19]. The scale contains 23 items and has four dimensions, namely, exhaustion in parenting, contrast with the previous parental self, feelings of being fed up, and emotional distancing. Responses were obtained on a 7-Point Likert scale ranging from 1 (never) to 7 (every day). Higher scores indicated higher levels of parental burnout. The scale has been validated to have good reliability and validity [19], and the Cronbach’s α in this study was 0.95.

#### Edinburgh postnatal depression scale (EPDS)

The EPDS was developed and revised by Cox et al. [20]. The scale contains 10 items. Each item was scored from 0 to 3. Higher summed scores indicated increased severity of postnatal depression. The EPDS has been widely used and has good reliability and validity [21]. The Cronbach’s α in this study was 0.86.

#### Covariates

The self-administered general information questionnaire includes the maternal age, infant’s age, maternal education level, marital status, average monthly family income, family residence, current work status, parity, difficulty in breastfeeding, and perceived parenting support from elders. Previous studies have shown that infants with self-regulation problems (e.g., sleep problems and crying) may be more likely to suffer from poor maternal-infant relationships, which might be associated with parental burnout [22]. Therefore, this study further adjusted for the difficult infant temperament. Difficult infant temperament was measured using the Parenting Stress Index-Short Form subscale (Difficult Child Temperament) [23]. The subscale is self-reported by the assessor and has a total of 5 items. Each item is scored on a 5-point Likert scale, with 1 indicating strong disagreement and 5 indicating strong agreement. The higher the total score of the items summed, the higher the degree of parenting difficulties of the offspring. The scale has been validated to have good reliability and validity [23], and the Cronbach’α in this study was 0.91.

### Statistical analyses

Statistical analyses were conducted using IBM SPSS software (v26.0) and Mplus 8. Frequencies and percentages were calculated for the categorical variables. Means and standard deviations were used to summarize continuous variables. Prior to conducting a multiple linear regression, a Pearson correlation analysis was performed to explore the relationship between postnatal depressive symptoms and parental burnout. Multiple linear regression was conducted to examine the association between postnatal depressive symptoms and the four dimensions of parental burnout after controlling for covariates.

This study was interested in examining the types of naturally occurring latent classes of parental burnout indicators that could be identified. Latent class analyses for different latent groups were conducted and the fit indices and class frequencies were compared. The variances were estimated to be equal between the classes. The estimation was performed step-by-step, starting from a one-class solution to estimate the parameters for 2, 3, …, k-class solutions. The solution that best fitted the data in accordance with the indicators, and that was deemed reasonable in terms of interpretation was chosen as the final latent class model. The model fitting indexes included the Akaike Information Criterion (AIC), Bayesian Information Criterion (BIC), Adjusted Bayesian Information Criteria (aBIC), Lo-Mendell-Rubin test (LMR), Bootstrap Likelihood Ratio Test (BLRT), and entropy. Binary logistic regression analysis was used to explore the differences between the latent classes of parental burnout in terms of postnatal depressive symptoms. In the sensitivity analysis, the scores of the four dimensions of parental burnout were dichotomized to further explore the robustness of the relationship between postnatal depressive symptoms and parental burnout using a binary logistic regression. As there was no definite cut-off value for the four dimensions of parental burnout, the mean plus 1.5 standard deviations was considered positive [24]. Statistical significance was set at two-sided, with *a p*-value of < 0.05.

## Results

### Characteristics of mothers in the study

In total, 583 mothers responded to the survey. Among them, mothers who did not answer key variables (postnatal depressive symptoms and parental burnout) were excluded, and 560 participants were finally included in the analysis.

The mean age of women was 30.8 ± 4.8 years and the mean age of these infants was 9.0 ± 3.2 months; the mean score for maternal postnatal depressive symptoms was 9.4 ± 5.0. The scores and symptom prevalence rates for the four dimensions of parental burnout are shown in Table 1. Pearson’s correlation analysis revealed a positive correlation between postnatal depressive symptoms and exhaustion in parenting (*r* = 0.495, *p* < 0.001) and contrast (*r* = 0.434, *p* < 0.001), and feelings of being fed up (*r* = 0.358, *p* < 0.001) and distance (*r* = 0.348, *p* < 0.001).

**Table 1:** The characteristics of the sample in the study(n = 560)

	Mean/Frequency	Standard Deviation/Percentage
Mother’s age(year)	30.8	4.8
Mother’s BMI(kg/m2)	22.5	3.4
Marital status		
married	536	95.7
others	23	4.1
NA	1	0.2
Current Residence		
Rural	33	5.9
county	49	8.8
city	477	85.2
NA	1	0.2
Education		
Junior high school and below	48	8.6
High school	157	28
Undergraduate	321	57.3
Postgraduate and above	29	5.2
NA	5	0.9
Household income		
Less than 4000 RMB	47	8.4
4001 ~ 6000 RMB	132	23.6
Above 6001RMB	366	65.4
NA	15	2.7
Parity		
Primipara	252	45
multipara	308	55
Employment status		
Normal work	297	53
Choose easy job to take care of your children	83	14.8
Full-time childcare	146	26.1
NA	34	6.1
Exchange parenting knowledge with other mothers		
Yes	501	89.5
No	51	9.1
NA	8	1.4
Overall breastfeeding difficulty		
Very simple	118	21.1
simple	208	37.1
No difficulty	106	18.9
A little difficult	90	16.1
Very difficult	34	6.1
NA	4	0.7
Parenting support from elders		
Not helping at all	17	3
Cannot help	52	9.3
Moderate help	152	27.1
Helpful	208	37.1
Great help	128	22.9
NA	3	0.5
Postnatal depressive symptoms	9.4	5
Infant’s age(month)	9	3.2
difficult infant temperament	12.2	4.4
Parental burnout	Mean(Standard Deviation)	prevalence (%)
exhaustion	9.9(9.0)	9.5
Contrast	5.3(6.0)	9.1
feelings of being fed up	2.7(4.6)	9.3
emotional distancing	2.0(3.0)	9.3

Abbreviations: BMI = Body mass index; NA = Not Available

Abbreviations: A1: exhaustion in parenting, A2: contrast with previous parental self, A3: feelings of being fed up, A4: emotional distancing. CLASS 1: C1 High PB; CLASS 2: C2 Low PB

### Associations between postnatal depressive symptoms and parental burnout

As shown in Table 2, postnatal depressive symptoms are positively associated with the four dimensions of parental burnout (all *p* < 0.05) after controlling for covariates. The results of the sensitivity analysis also indicated that postnatal depressive symptoms were associated with different dimensions of parental burnout (See Supplementary Table 2).

**Table 2:** Associations between postnatal depressive symptoms and each dimension of parental burnout

	*B*	*SE*	*β*	*t*	*R*	*R* ^*2*^
exhaustion in parenting ^a^	0.554	0.098	0.345	**5.629** ^***^	0.549	0.302
contrast with previous parental self ^a^	0.418	0.073	0.365	**5.693** ^***^	0.486	0.236
feelings of being fed up ^a^	0.179	0.056	0.214	**3.165** ^**^	0.387	0.150
emotional distancing ^a^	0.159	0.036	0.292	**4.406** ^***^	0.426	0.182
parental burnout ^a^	1.266	0.228	0.350	**5.556** ^***^	0.510	0.260

*Note*: ^a^: The estimates were adjusted for maternal age, marital status, education, household income, birth, employment status, infant age, difficult infant temperament, exchange of parenting knowledge with other mothers, difficulty in breastfeeding, parenting support from elders, and infant sex.

^**^, *P*<0.01; ^***^, *P*<0.001

### Latent categories of parental burnout and comparison of different categories

As shown in Table 3, a comparison of the fit indices and class frequencies showed that the model fit indices were the worst when the number of latent classes was set to 1 (zero models). When a second group was included in the analyses, the BIC, aBIC, and AIC slightly decreased and the entropy value slightly increased compared to the three profile solutions, and the class sizes were acceptable. The p-values of LMR and BLRT of the two-category model were significant, indicating that the two-category model was better than the one-category model. In the three-category model, the p-value of LMR of the three-category model was not significant, indicating that the three-category model was not superior to the two-category model. Therefore, because the two-category model was theoretically meaningful, and the goodness-of-fit indices indicated that the second latent group was necessary, the two-latent-group solution was considered the best model. The mean probability of attribution for each latent class ranges from 0.988 to 0.998, indicating that this classification is plausible.

**Fig. 1:**
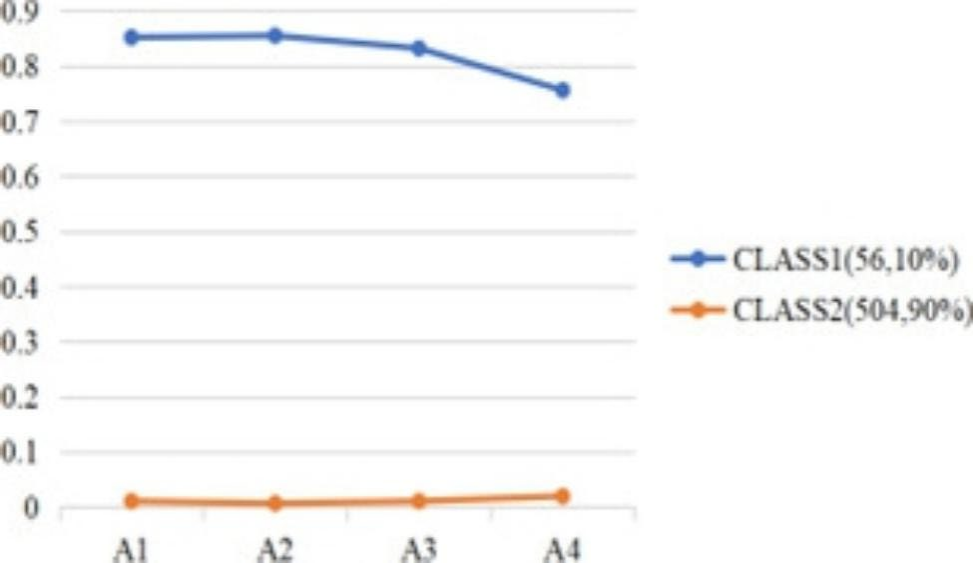
Latent classes on four dimensions of parental burnout

The first latent class, 90% of the mothers, was characterized by a low level of all parental burnout components. The second latent class (10% of the parents) was characterized by a relatively high level of all parental burnout components. The latent classes were labeled low parental burnout (i.e., low PB) and high parental burnout (i.e., high PB). High PB scores were higher than low PB scores in all dimensions (See Table 4; Fig. 1).

The binary logistic regression model showed that postnatal depressive symptoms were an influential factor for the different subtypes of parental burnout (*OR =* 1.15, 95% CI:1.09 ~ 1.22, *p* < 0.001, Model 1). This result remained significant after controlling for confounding factors (*OR* = 1.12, 95% CI: 1.03 ~ 1.23, *p* = 0.011, Model 2) (See Supplementary Table 1).

**Table 3:** Fitting information for a model of potential categories of parental burnout

Category	K	Loglikelihood	AIC	BIC	aBIC	Entropy	BLRT	LMR
1	4	-692.36	1392.72	1410.032	1397.334	-	-	-
2	9	-400.918	819.836	858.787	830.217	0.983	<0.001	<0.001
3	14	-397.343	822.686	883.278	838.835	0.939	0.125	0.4283
4	19	-396.467	830.935	913.165	852.85	0.902	0.500	0.1472

Note: K: difference in the number of parameters; AIC = Akaike Information Criterion; BIC = Bayesian Information Criterion; aBIC = Adjusted Bayesian Information Criterion; BLRT = Bootstrap Likelihood Ratio Test; LMR = Lo-Mendell-Rubin test

**Table 4:** Comparison of latent classes on four dimensions of parental burnout

	C1 High PB	C2 Low PB	*t*
EX	29.86 ± 7.39	7.66 ± 5.94	25.836^***^
CO	19.04 ± 5.83	3.76 ± 3.55	19.210^***^
FU	13.89 ± 5.52	1.45 ± 2.22	16.728^***^
ED	8.52 ± 3.35	1.26 ± 1.94	15.911^***^

Abbreviations: EX = exhaustion in parenting; CO = contrast with previous parental self; FU = feelings of being fed up; ED = emotional distancing; High PB = high parental burnout; Low PB = low parental burnout

^***^, *P*<0.001

## Discussion

Parental burnout is attracting the attention of researchers because of its pervasive consequences. To the best of our knowledge, this study is the first to focus on one of the vulnerable groups, postnatal women. This study found that the prevalence of the four burnout dimensions was 9.5% (exhaustion in parenting), 9.1% (contrast with previous parental self), 9.3% (feelings of being fed up), and 9.3% (emotional distancing). Additionally, the relationships between postnatal depressive symptoms and each dimension of parental burnout at both population and individual levels were examined using multiple methods.

At the population level, postnatal depressive symptoms were positively associated with all dimensions of parental burnout, which was also verified by sensitivity analysis. This finding was consistent with previous results drawn from studies of mothers raising older children [1, 13], which also found a close association between postnatal depressive symptoms and parental burnout.

First, the association was analyzed from the individual perspective by using person-oriented research (LCA), making it possible to identify distinct homogenous classes of mothers who suffer from parental burnout. Two categories were found, namely, the low PB class, characterized by a low level of all components of parental burnout, and the high PB class, characterized by a relatively high level of all components of parental burnout. These results are partially consistent with previous research showing that approximately 85.7% of Finnish parents reported low scores on different dimensions of parental burnout and 8% reported high scores on three dimensions of parental burnout, except for a low level of emotional distancing. Furthermore, a third profile was characterized by a high level of emotional distancing from one’s children and an average level of parental exhaustion, in contrast with the previous self, and feelings of fed up were computed [16]. These differences may be because the study was conducted in Finnish on parental burnout among fathers and mothers, whereas the present study focused only on parental burnout in postpartum mothers. The study results suggest that parental burnout symptoms manifest differently among postpartum mothers, and that mothers in the high parental burnout group had higher depression scores than those in the low parental burnout group. A person-centered approach facilitates the identification of individual differences for individualized interventions.

The results of this study suggest a close association among postnatal women. It may be because the postpartum period refers to a specific period in a mother’s life that can increase the risk of mental health problems [8]. Mothers with high postpartum depression scores are in an unstable mental state and may be prone to negative emotions, such as self-denial, when dealing with parenting-related issues. Furthermore, they are reluctant to solve child-related problems, stay away from their children both psychologically and behaviorally, and are more prone to parenting burnout. This study indicated that the postpartum period should be a sensitive window for intervention because of the high prevalence of maternal depression and burnout at this stage. Their close relationship suggests that postnatal depressive symptoms may be an indicator of parental burnout. Therefore, mothers with postnatal depressive symptoms should be closely screened for parental burnout. Intervention programs targeting postnatal depressive symptoms are promising because they may be effective in improving parental burnout and, therefore, bring great benefits.

Despite these potentials and values, this study still has some limitations. First, postnatal depressive symptoms and parental burnout in the study were collected through a self-assessment questionnaire, it may be subject to recall bias and social desirability bias. In addition, mothers included in this study were mainly recruited from a tertiary hospital in one city (Jinan, Shandong Province), mostly lived in the city and were well-educated, which limited the generalizability of the findings. In addition, this study was a cross-sectional study and cannot disclose the causal relationship between postpartum depressive symptoms and parental burnout in mothers.

## Conclusion

In this study, the prevalence of parental burnout in the first year postpartum is approximately 10%. The postnatal depressive symptoms were positively associated with parental burnout, and this association is consistent at both individual and population levels. These results suggest that decreasing postnatal depression symptoms may be a possible way to prevent parental burnout in postnatal mothers. Some intervention programs such as meditation and cognitive behavioral therapy [25, 26], have been proven to be effective in reducing postnatal depressive symptoms. Based on our results, those with parental burnout may also benefit from these available intervention programs for postnatal depression. Further studies are needed to examine this assumption.

## Electronic supplementary material

Below is the link to the electronic supplementary material.

Supplementary Material 1

## Data Availability

The datasets generated and/or analysed during the current study are not publicly available due maternal privacy implications but are available from the corresponding author on reasonable request.
