# Supplementary material for: Exploring the relationship between postnatal depressive symptoms and parental burnout from the perspective of the population and individual level
Source: BMC Psychiatry. 2023 Jun 7;23:409. doi: 10.1186/s12888-023-04853-2 (PMC10246355; doi:10.1186/s12888-023-04853-2)
Supplement: Supplementary file 1 — Supplementary Material 1 [file 12888_2023_4853_MOESM1_ESM.docx]

Supplementary Table 1. Binary logistic regression of two latent classes of parental burnout

|  | *β* | Wald | *OR*（95%CI） | *p* |
| --- | --- | --- | --- | --- |
| Model1 |  |  |  |  |
| PDS | 0.141 | 21.346 | 1.15(1.09~1.22) | <0.001 |
| Model2^a^ |  |  |  |  |
| PDS | 0.116 | 6.399 | 1.12(1.03~1.23) | 0.011 |

Abbreviations: OR: Odds Ratio, PDS: postnatal depressive symptoms.

^a^: The estimates were adjusted for maternal age, marital status, education, household income, birth, employment status, infant age, difficult infant temperament, exchange of parenting knowledge with other mothers, difficulty in breastfeeding, parenting support from elders, and infant sex.

Supplementary Table 2. Binary logistic regression analysis of the associations between postnatal depressive symptoms and each dimension of parental burnout

| Variables | *β* | Wald | *OR*（95%CI） | *p* |
| --- | --- | --- | --- | --- |
| Model 1^a^ |  |  |  |  |
| PDS | 0.146 | 17.386 | 1.16(1.08~1.24) | <0.001 |
| Model 2^b^ |  |  |  |  |
| PDS | 0.125 | 12.063 | 1.13(1.06~1.22) | 0.001 |
| Model 3^c^ |  |  |  |  |
| PDS | 0.120 | 12.176 | 1.13(1.05~1.21) | <0.001 |
| Model 4^d^ |  |  |  |  |
| PDS | 0.124 | 12.676 | 1.13(1.06~1.21) | <0.001 |

Abbreviations: OR: Odds Ratio, PDS: postnatal depressive symptoms. The estimates were adjusted for maternal age, marital status, education, household income, birth, employment status, infant age, difficult infant temperament, exchange of parenting knowledge with other mothers, difficulty in breastfeeding, parenting support from elders, and infant sex.

^a^: Dependent variable was exhaustion in parenting;

^b^: Dependent variable was contrast with previous parental self;

^c^: Dependent variable was feelings of being fed up;

^d^: Dependent variable was emotional distancing.
